# Validation of a deep learning-based AI model for breast cancer risk stratification in postmenopausal ER+/HER2-breast cancer patients

**DOI:** 10.1016/j.breast.2025.104671

**Published:** 2025-12-04

**Authors:** Sandra Sinius Pouplier, Abhinav Sharma, Pekka Ruusuvuori, Johan Hartman, Maj-Britt Jensen, Bent Ejlertsen, Mattias Rantalainen, Anne-Vibeke Lænkholm

**Affiliations:** a Department of Surgical Pathology, Zealand University Hospital, Sygehusvej 10, 4000, Roskilde, Denmark; b Department of Clinical Medicine, Faculty of Health and Medical Sciences, University of Copenhagen, Blegdamsvej 3B, 2200, Copenhagen N, Denmark; c Department of Medical Epidemiology and Biostatistics, Nobels Väg 12A, 171 77 Stocholm, Karolinska Institute, Sweden; d Institute of Biomedicine, University of Turku, Kiinamyllynkatu 10, 20520, Turku, Finland; e Department of Oncology-Pathology, Karolinska Institute, Solnavägen 30, 171 64 Solna, Sweden; f Danish Breast Cancer Group, Department of Oncology, Centre for Cancer and Organ Disease, Rigshospitalet, Blegdamsvej 9, Copenhagen University Hospital, 2100, Copenhagen, Denmark; g Department of Oncology, Centre for Cancer and Organ Disease, Rigshospitalet, Blegdamsvej 9, Copenhagen University Hospital, Denmark

**Keywords:** Breast cancer, Prognostication, AI-models, Deep learning, Stratipath breast, Survival analysis

## Abstract

**Background:**

Breast cancer prognostication is crucial for treatment decisions, and the Nottingham Histologic Grade (NHG) system is widely used. However, NHG suffers from interobserver variability, and its division into three risk groups leaves the intermediate group (comprising ∼50 % of patients) overrepresented, making individualized treatment planning challenging as prognosis within this group differ widely.

**Objectives:**

This study aimed to validate the prognostic value of Stratipath's low and high-risk categories and five risk groups and compare NHG performance with the Stratipath deep-learning-based model.

**Methods:**

We analyzed clinical data from 2466 postmenopausal, ER+/HER2-breast cancer patients who did not receive chemotherapy according to guidelines at that time. The NHG and Stratipath models were compared using concordance index and hazard ratios (HR) for distant recurrence (DR), with time to any recurrence (TR) and overall survival (OS) as secondary endpoints.

**Results:**

The Stratipath five-risk group model showed similar performance to the NHG-system in predicting DR (c-index 0.71 vs. 0.72). HR for DR for Stratipath risk groups 2, 3, 4, and 5 were 1.91 (95 % CI: 1.17–3.13), 2.63 (95 % CI: 1.63–4.24), 3.18 (95 % CI: 2.00–5.07), and 3.25 (95 % CI: 2.00–5.28), respectively (p < 0.0001). In the NHG 2 subgroup, Stratipath Breast retained prognostic value for DR (HR for groups 3–5 vs. group 1: 1.73–1.85; p = 0.05), with a c-index of 0.71.

**Conclusions:**

The Stratipath AI model performs similarly to the NHG system. Further prospective validation of the clinical benefits of differentiating Stratipath risk groups 2 and 3 in treatment strategies would be valuable.

## Highlights

• Stratipath Breast AI model externally validated in 2466 ER+/HER2− patients from the DBCG99C cohort.
• AI model performed comparably to NHG for predicting distant recurrence (c-index 0.71 vs. 0.72).
• Stratipath five-group model provided prognostic value within the NHG2 intermediate-risk subgroup.
• Validation supports AI grading as a potential tool to enhance reproducibility in breast cancer risk stratification.

## Abbreviations

NHG: Nottingham histological grade
NHG1: Nottingham histological grade 1
NHG2: Nottingham histological grade 2
NHG3: Nottingham histological grade 3
HR: Hazard ratio
DR: Distant recurrence
TR: Time to any recurrence
OS: Overall survival
MDT: Multidisciplinary team
AUC: Area under the curve
BC: Breast cancer
IHC: Immunohistochemistry
H&E: Hematoxylin and eosin
CIF: Cumulative incidence function
KM: Kaplan-Meier
c-index: Concordance index
ER: Estrogen Receptor
HER2: Human epidermal growth factor receptor 2
PFS: Progression-free survival

## 1 Introduction

Pathology departments worldwide are undergoing a digital transformation, shifting from traditional slide assessment under the microscope to digital pathology. This transition enables improved collaboration among pathologists and the integration of new technologies [1]. Precise cancer treatment is essential to improve survival while minimizing the risk of overtreatment, which can lead to long-term adverse effects and negatively impact the quality of life for long-term survivors.

When a patient is diagnosed with breast cancer (BC), multidisciplinary team (MDT) conferences decide on an individualized treatment plan. These discussions aim to select the most appropriate treatment for each patient, considering numerous variables that influence patient outcomes. The pathological evaluation of tumor malignancy grade plays a central role in determining the treatment strategy.

For malignancy grading, the Nottingham Histological Grading (NHG) system is widely used. It is based on histological characteristics in tumors that are associated with prognosis in breast cancer: gland formation, nuclear pleomorphism and mitotic rate. In the 1990's the NHG system was implemented, after Elston and Ellis [2] revised the original histological assessment from Bloom and Richardson [3], making it more quantitative and reproducible. NHG is validated as an independent prognostic factor, as demonstrated in several studies [[4], [5], [6]].

However, the NHG system still faces reproducibility issues, with significant variations in inter-observer agreement across studies using both light microscopy and digital pathology [7,8]. Inter-observer variability highlights the lack of objectivity in the current methods. Thus, there is a need to investigate supplementary methods in breast cancer pathology to increase objectivity and reproducibility.

### 1.1 Pathomics – AI in pathology

Deep learning-based AI-models show great potential to enhance accuracy and efficiency in pathology [9,10]. In addition, AI-models is considered to hold considerable promise in addressing the reproducibility issues in histological grading [4,11,12].

In AI technology, various strategies are employed for constructing models. One common approach is to utilize datasets annotated by expert pathologists [13]. However, as deep learning (DL) models take on more complex tasks, such as predicting prognosis from histomorphological patterns, reliance on annotated datasets may limit their ability to detect patterns beyond human perception. Notably, there have been examples demonstrating that DL technology can predict mRNA expression levels using HE-stained slides as input [14]. While annotations have the benefit of more control of the process, models trained in a weakly supervised manner may be better suited to address complex clinical applications by capturing patterns not restricted to predefined labels.

Findings suggest that, with sufficiently large datasets, classification models can achieve a very high area under the curve (AUC) score, enabling performance comparable to expert-level standards [15,16].

AI is expected to play a future role in patient-centered treatment plans, particularly for borderline cases, where AI models could help guide decisions on chemotherapy. AI-driven models represent a promising solution for improving diagnostic accuracy and guiding oncological treatment decisions [17], while the black-box nature of AI has raised concerns and a need for explainability to build trust and transparency. To address the issue of explainability in pathology models utilizing slide-level annotations, we believe that thorough external validation in datasets with sufficient follow-up time is the most effective approach [18]. Stratipath Breast do not offer results for pixel level interpretation, however in clinical usage, the report includes graphical representations of analyzed tissue areas, as well as areas excluded due to poor image quality, enabling the pathologist to confirm that relevant tissue areas were included in the analysis.

The Stratipath® Breast model is a CE-IVD-certified AI tool that extends the previously published DeepGrade model, originally developed using data from 1567 breast cancer patients [19]. Further details of the DeepGrade model have previously been described [19]. The initial model stratified patients into low- and high-risk groups. The commercialized Stratipath® model further assigns patients into five distinct risk groups based on a continuous risk score.

In this retrospective study, we aimed to evaluate the prognostic performance of the Stratipath Breast model in predicting distant recurrence (DR) and time to any recurrence (TR) at 10 years, as well as overall survival (OS) over 21 years of follow-up, in postmenopausal Estrogen Receptor (ER)-positive, Human Epidermal Growth factor Receptor 2 (HER2)-negative breast cancer cohort.

## 2 Methods

### 2.1 Patients

The DBCG99C cohort derive from the Danish Breast Cancer Group population-based database, which collects prospective and complete clinical data from all breast cancer patients in Denmark [20]. Further details on the DBCG99C cohort are available in prior publications [21,22].

The DBCG99C includes postmenopausal ER+/HER2- BC patients diagnosed 2000–2003. Patients were aged ≥50 years and met at least one of the following criteria: tumor size greater than 20 mm, ductal histology with malignancy grade 2 or 3, or the presence of 1–3 positive lymph nodes.

ER expression was assessed using immunohistochemistry (IHC) with a 10 % cut-off level.

Patients were screened for distant metastases through physical examination and chest radiography, with additional imaging (bone scintigraphy or radiography) for those with bone pain or elevated resorption markers. Treatment included breast-conserving surgery or mastectomy with sentinel node biopsy, followed by axillary dissection, if node-positive disease. Endocrine therapy (tamoxifen or an aromatase inhibitor) was prescribed for minimum 4.5 years.

Radiotherapy (48 Gy in 2 Gy fractions, five times per week) was administered to the residual breast or chest wall for patients under 70 with tumors >50 mm and to regional lymph nodes in node-positive patients. TNM status and AJCC 8th edition prognostic stage were derived from available clinicopathological data (tumor size, lymph node status, grade, and receptor status).

### 2.2 Application of Stratipath Breast for analysis of the DBCG99C cohort

**Fig. 1:**
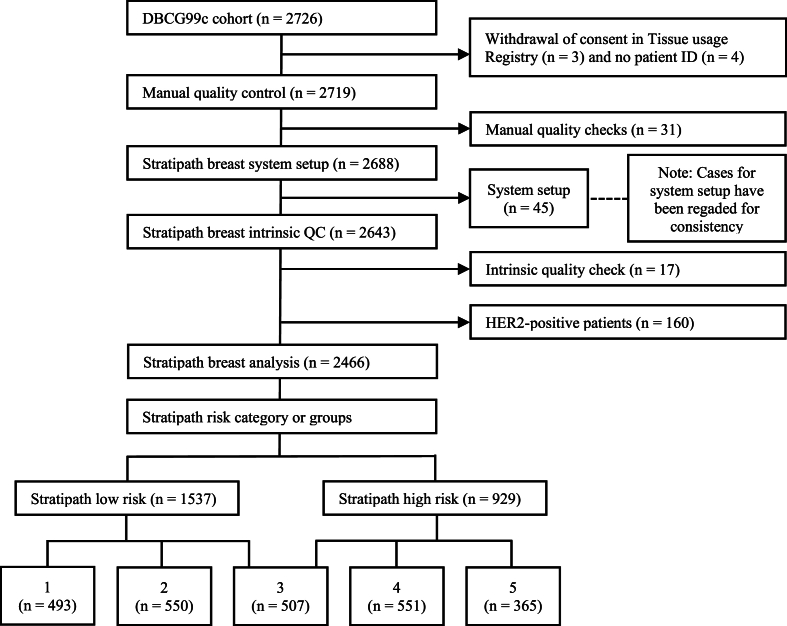
Patient flowchart of Stratipath Breast model applied on DBCG99C, Note: Cases for system setup have been regraded for consistency.

We applied the Stratipath® breast model to the DBCG99C cohort (n = 2466). Formalin-fixed paraffin-embedded (FFPE) tissue blocks were sectioned and stained with hematoxylin and eosin (H&E) at the same lab facility. The H&E-stained slides were digitized at 40× magnification using either the Hamamatsu NanoZoomer XR or NanoZoomer S360 (Hamamatsu Photonics). All slides were reviewed in low resolution to manually assess stain intensity, which correlates to the thickness of the slides. If the slides were too thick to properly see the tissue and cell structure, it was excluded (n = 31). 45 slides consisting of 15 slides from each NHG 1, 2 and 3 were used for system setup according to the standard use of Stratipath Breast and were excluded from the analysis. The built-in quality control excluded 17 slides (0,6 %) (Fig. 1).

### 2.3 Endpoints

In this study, we have used the primary endpoint DR, defined as time from breast cancer surgery to distant metastasis or death from breast cancer. Secondary endpoints are TR and OS. TR is defined as time from breast cancer surgery to local recurrence, distant recurrence or death from breast cancer. OS is defined as time from breast cancer surgery to death from any cause. For OS, follow-up was linked to the Danish Central Population Registry until 2024.02.01.

In distant recurrence and time to any recurrence, we consider competing events to be contralateral breast cancer, other malignancies, and death from other causes.

### 2.4 Statistical analysis

Univariable and multivariable analyses were performed for DR, TR and OS. Cumulative incidence function (CIF) plots were generated for DR and TR, while Kaplan-Meier (KM) plots were created for OS. Gray's test and the log-rank test was applied for CIF/KM estimates, respectively. Follow-up time was estimated using the inverse KM method.

For DR and TR, competing risk analysis was performed using the Fine-Gray proportional subdistribution hazard model. In OS, the Cox proportional hazards model was used. Baseline characteristics were compared across Stratipath risk groups using the Chi-squared test. In the multivariable analysis we included age (continuous), tumor size (continuous), number of positive lymph nodes (continuous) and ER expression (continuous, defined as the percentage of positive tumor cells). Stratipath risk categories (low vs. high) or risk groups (categorical 1,2,3,4 and 5) and NHG-status (categorical 1,2, and 3) are included in separate models. In the dataset, 32 patients had ER status recorded as “Positive” due to the absence of quantitative information on ER status (Table 1A). To incorporate these patients into the analysis, we assigned them an ER status of 100 %, which represents the most probable value. Continuous variables were evaluated for non-linearity using martingale residuals. Based on this evaluation, the tumor size variable was log-transformed for endpoints DR and TR, while no transformations were necessary for the other continuous variables. Models were fitted for all patients and a subgroup analysis was conducted for Nottingham histological grade 2 (NHG2) patients (n = 1493). The proportional hazards assumption was assessed using Schoenfeld's global test and visually by Schoenfeld residuals. In the Fine-Gray models for DR and TR, the hazard ratio (HR) for ER status and Stratipath risk categories (low vs. high) were found to be non-proportional over the 10-year follow-up period. Therefore, models for DR and TR incorporated these variables as time-dependent, with separate estimates for intervals before and after 5 years. In the NHG2 subgroup analysis, only ER status was found to be non-proportional and split at 5 years. In the Cox PH model, variables Stratipath risk category (low, high), tumorsize and ER was modeled for two time periods with split at 5 years, and age was modeled with cutpoints at 5 and 10 years. The NHG-system was compared with the Stratipath Breast model based on concordance index (c-index) [23]. Analysis were performed in R v4.4.1.

**Table 1A:** Patient baseline characteristics.

Variable	Stratipath risk groups, n (%)					Total	p-value
	1	2	3	4	5	n = 2466	
Age							0.02
50-59	203 (41.2)	209 (38.0)	179 (35.3)	194 (35.2)	120 (32.9)	905 (36.7)	
60-69	210 (42.6)	228 (41.5)	203 (40.0)	228 (41.4)	174 (47.7)	1043 (42.3)	
≥ 70	80 (16.2)	113 (20.5)	125 (24.7)	129 (23.4)	71 (19.5)	518 (21.0)	
Positive lymph nodes							<0.0001
0	180 (36.5)	230 (41.8)	236 (46.5)	282 (51.2)	193 (52.9)	1121 (45.5)	
1	187 (37.9)	184 (33.5)	134 (26.4)	156 (28.3)	86 (23.6)	747 (30.3)	
2	84 (17.0)	97 (17.6)	82 (16.2)	69 (12.5)	51(14.0)	383 (15.5)	
3	42 (8.5)	39 (7.1)	55 (10.8)	44 (8.0)	35 (9.6)	215 (8.7)	
Tumor size (mm)							0.02
≤ 10	69 (14.0)	51 (9.3)	45 (8.9)	42 (7.6)	23 (6.3)	230 (9.3)	
11-20	210 (42.6)	238 (43.3)	219 (43.2)	237 (43.0)	144 (39.5)	1048 (42.5)	
21-30	146 (29.6)	185 (33.6)	166 (32.7)	196 (35.6)	136 (37.3)	829 (33.6)	
> 30	68 (13.8)	76 (13.8)	77 (15.2)	76 (13.8)	62 (17.0)	359 (14.6)	
Nottingham Grade							<0.0001
1	265 (53.8)	179 (32.5)	124 (24.5)	78 (14.2)	20 (5.5)	666 (27.0)	
2	220 (44.6)	352 (64.0)	347 (68.4)	374 (67.9)	200 (54.8)	1493 (60.5)	
3	8 (1.6)	19 (3.5)	36 (7.1)	99 (18.0)	145 (39.7)	307 (12.4)	
Estrogen Receptor							0.0004
10-59	39 (7.9)	39 (7.1)	64 (12.6)	57 (10.3)	54 (14.8)	253 (10.3)	
60-89	100 (20.3)	117 (21.3)	87 (17.2)	134 (24.3)	74 (20.3)	512 (20.8)	
90-99	132 (26.8)	141 (25.6)	122 (24.1)	139 (25.2)	112 (30.7)	646 (26.2)	
100	216 (43.8)	246 (44.7)	229 (45.2)	214 (38.8)	118 (32.3)	1023 (41.5)	
Positive∗	6 (1.2)	7 (1.3)	5 (1.0)	7 (1.3)	7 (1.9)	32 (1.3)	
TNM∗∗							<0.0001
T1N0M0	68 (13.8)	110 (20.0)	120 (23.7)	140 (25.4)	100 (27.4)	538 (21.8)	
T1N1M0	211 (42.8)	179 (32.5)	144 (28.4)	139 (25.2)	67 (18.4)	740 (30.0)	
T2N0M0	103 (20.9)	114 (20.7)	114 (22.5)	139 (25.2)	89 (24.4)	559 (22.7)	
T2N1M0	98 (19.9)	133 (24.2)	116 (22.9)	123 (22.3)	104 (28.5)	574 (23.3)	
T3N0M0	9 (1.8)	6 (1.1)	2 (0.4)	3 (0.5)	4 (1.1)	24 (1.0)	
T3N1M0	4 (0.8)	8 (1.15)	11 (2.2)	7 (1.3)	1 (0.3)	31 (1.3)	
Stage∗∗							<0.0001
I	68 (13.8)	110 (20.0)	120 (23.7)	140 (25.4)	100 (27.4)	538 (21.8)	
II	412 (83.6)	426 (77.5)	374 (73.8)	401 (72.8)	260 (71.2)	1873 (76.0)	
III	13 (2.6)	14 (2.5)	13 (2.6)	10 (1.8)	5 (1.4)	55 (2.2)	

Table 1A. Number of patients (%).

∗ At least 10 % of the exact ER expression level percentages are unknown.

∗∗ TNM categories and prognostic stage were defined according to AJCC 8th edition.

## 3 Results

This study evaluated the prognostic performance of the Stratipath Breast model in the DBCG99C cohort. The median follow-up time was 8.9 years for DR and TR, and 21.9 years for OS.

The study included 2466 patients stratified into both low and high risk, and risk groups 1–5 according to the Stratipath classification. There were 279 DR events, 307 TR events, and 1472 deaths by any cause. Age distribution varied significantly across risk groups (p = 0.02), with younger patients in lower-risk and older patients in higher-risk groups. Lymph node involvement also differed significantly (p < 0.0001), with higher-risk groups showing a greater number of positive lymph nodes. Tumor size distribution followed a similar pattern (p = 0.02), as smaller tumors were more common in lower-risk groups, while larger tumors were more prevalent in higher-risk categories. NHG correlated strongly with Stratipath risk (p < 0.0001); with NHG1 in lower and NHG3 in high-risk groups. Lower ER expression was associated with higher risk-groups (p = 0.0004). Overall, lower-risk groups showed favorable clinicopathological features, whereas higher-risk groups had more aggressive tumor characteristics (Table 1A).

### 3.1 Stratipath risk categories low and high

In the low and high-risk model, 62.3 % (n = 1537) of participants were classified as low risk, while 37.7 % (n = 929) were classified as high risk (Fig. 1).

**Fig. 2:**
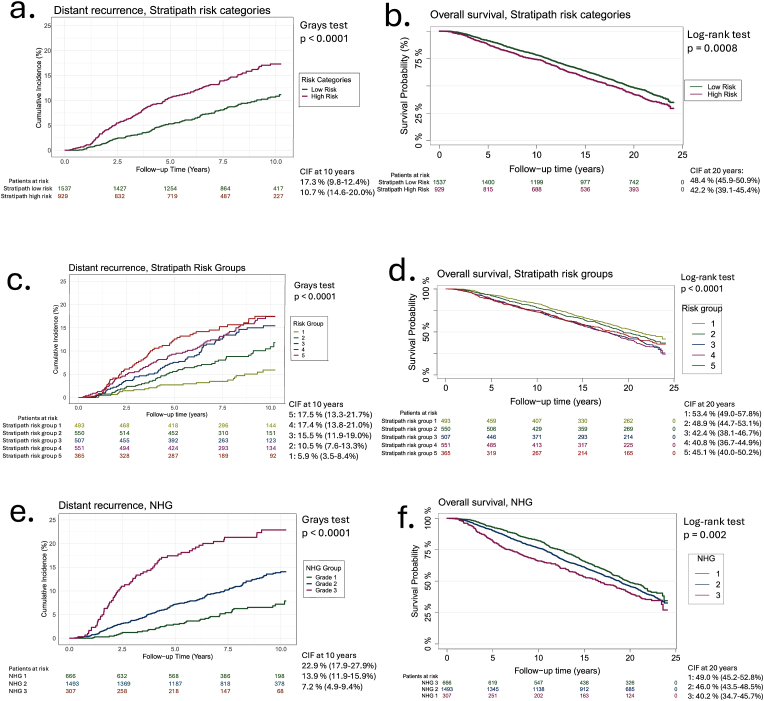
Univariable analysis for DR and OS CIF and KM plots for DR and OS. a) CIF plot for DR across Stratipath risk categories, b) KM plot for OS across Stratipath risk categories, c) CIF plot for DR across Stratipath risk groups, d) KM plot for OS across Stratipath risk groups, e) CIF plot for DR across NHG grades, f) KM plot for OS across NHG grades. The CIF plots show cumulative incidences at 10 years (95 % CI), while the KM plots display survival probabilities at 20 years (95 %CI).

We found a significant difference in DR over time between the Stratipath low- and high-risk categories (Grays test, p < 0.0001). A similar pattern was observed for OS rates over time (log-rank test, p = 0.0008) (Fig. 2a and b),

In the multivariable model examining DR, we found a significant prognostic effect of the Stratipath risk categories. Specifically, the high-risk group showed an adjusted subdistribution HR of 2.01 (95 % CI: 1.49–2.71) for DR during the first 5 years (Table 2), and the effect of the cumulative incidence is likewise seen in Fig. 2A, indicating that patients in the high-risk group had more than twice the risk of distant recurrence within the first five years.

We also observed significant prognostic effects when assessing TR and OS. In OS, the high-risk group exhibited an adjusted HRs of 1.35 (95 % CI: 1.05–1.73) during the first 5 years. However, for the period beyond 5 years, the HRs for the high-risk group were not significantly higher compared to the low-risk group, indicating no continued statistical difference in OS beyond the 5-year mark.

When performing subgroup analysis for NHG2 patients, we did not find significant differences between the low and high-risk group in either time to DR, TR or OS.

### 3.2 Stratipath 5-risk groups

In the 5 risk groups, we see that group 1 constitutes 20 % (n = 493), risk group 2 constitute of 22 % (n = 550), risk group 3 of 21 % (n = 507), risk group 4 of 22 % (n = 551) and risk group 5 of 15 % (n = 365), respectively. We found a significant difference in DR among the 5 risk groups (Grays test, p < 0.0001) and OS (log-rank test, p < 0.0001) between the 5 risk groups (Fig. 2c and d). In the CIF plot for DR (Fig. 2c), the estimated cumulative incidence curves for risk group 3 ends slightly below those for groups 4 and 5, indicating a marginally lower cumulative incidence, however formal testing did not suggest any violation of the proportional hazards assumption. In contrast, risk groups 1 and 2 maintain distinct slopes throughout the follow-up period, reflecting consistently lower risk progression.

In the adjusted HRs, we observed a statistically significant difference across risk groups 1 to 5 for DR, TR, and OS (Table 2). The HRs increased progressively with the risk category, confirming a strong association between risk stratification and outcomes. Notably, risk groups 4 and 5 had similar adjusted HRs across all endpoints, suggesting a potential plateau effect in higher-risk groups as seen in the univariable analysis (Fig. 2e and f).

The concordance index (c-index) for the Stratipath risk group models for DR, TR, and OS was 0.71, 0.68, and 0.66, respectively, indicating a moderate discriminative ability of the model across all endpoints.

Discordant cases were defined as NHG3 classified as Stratipath risk group 1 (*discordant low*) and NHG1 classified as Stratipath risk group 5 (*discordant high*). Among discordant low patients (n = 8), most were Luminal A (7/8), while discordant high patients (n = 20) were predominantly Luminal B (15/19) (Table 1B). Events were rare, with one DR in the discordant high group and none in the discordant low group.

**Table 1B:** Patient baseline characteristics for discordant patients.

Variable	Discordant risk groups, n (%)		Total	p-value
	Low (n = 8)	High (n = 20)	(n = 28)	
Age				0.71
50-59	2 (25.0)	8 (40.0)	10 (35.7)	
60-69	3 (37.5)	5 (25.0)	8 (28.6)	
≥ 70	3 (37.5)	7 (35.0)	10 (35.7)	
Positive lymph nodes				0.09
0	4 (50.0)	4 (20.0)	8 (28.6)	
1	3 (37.5)	3 (15.0)	6 (21.4)	
2	1 (12.5)	7 (35.0)	8 (28.6)	
3	0 (0.0)	6 (30.0)	6 (21.4)	
Tumor size (mm)				0.32
≤ 10	1 (12.5)	0 (0.0)	1 (3.6)	
11-20	2 (25.0)	9 (45.0)	11 (39.3)	
21-30	4 (50.0)	10 (50.0)	14 (50.0)	
> 30	1 (12.5)	1 (5.0)	2 (7.1)	
Nottingham Grade				NA
1	0 (0.0)	20 (100.0)	20 (71.4)	
2	0 (0.0)	0 (0.0)	0 (0.0)	
3	8 (100.0)	0 (0.0)	8 (28.6)	
Estrogen Receptor				0.34
10-59	1 (12.5)	1 (5.0)	2 (7.1)	
60-89	3 (37.5)	4 (20.0)	7 (25.0)	
90-99	1 (12.5)	9 (45.0)	10 (35.7)	
100	3 (37.5)	6 (30.0)	9 (32.1)	
Positive	0 (0.0)	0 (0.0)	0 (0.0)	
Molecular Subtype				0.004
Luminal A	7 (87.5)	4 (20.0)	11 (39.3)	
Luminal B	1 (12.5)	15 (75.0)	16 (57.1)	
BasalLike	0 (0.0)	0 (0.0)	0 (0.0)	
HER2Enriched	0 (0.0)	1 (5.0)	1 (3.6)	
ROR group				0.005
Low	3 (37.5)	0 (0.0)	3 (10.7)	
Intermediate	2 (25.0)	2 (10.0)	4 (14.3)	
High	3 (37.5)	18 (90.0)	21 (75.0)	
TNM∗∗				0.11
T1N0M0	2 (25.0)	0 (0.0)	2 (7.1)	
T1N1M0	1 (12.5)	9 (45.0)	10 (35.7)	
T2N0M0	2 (25.0)	4 (20.0)	6 (21.4)	
T2N1M0	3 (37.5)	7 (35.0)	10 (35.7)	
Stage∗∗				0.07
I	2 (25.0)	0 (0.0)	2 (7.1)	
II	6 (75.0)	20 (100.0)	26 (92.9)	

Table 1B. Number of patients (%). Discordant classifications were defined as discordant low (patients with Stratipath risk group 1 and NHG 3) and discordant high (patients with Stratipath risk group 5 and NHG 1).

∗At least 10 % of the exact ER expression level percentages are unknown.

∗∗ TNM categories and prognostic stage were defined according to AJCC 8th edition.

In the subgroup of patients with NHG2 tumors, the association between Stratipath risk groups for DR and TR was statistically significant (p = 0.05 for both endpoints). Compared to risk group 1 (reference), higher risk groups demonstrated increased HRs for DR and TR. For DR, risk groups 3 to 5 showed HRs of 1.85 (95 % CI: 1.06–3.20), 1.73 (0.99–3.00), and 1.73 (0.94–3.18), respectively. Similar trends were observed for TR. For OS, no statistically significant associations were observed across the risk groups (p = 0.31).

### 3.3 Nottingham risk grading

Table 1C (supplementary) shows the NHG distribution across the five Stratipath risk groups. NHG1 tumors were mainly in groups 1–2, NHG3 tumors in groups 4–5 and NHG2 tumors were distributed across groups 2–4 (23–25 % each). In the unadjusted results, we found a significant difference in DR among the NHG system groups (Grays test, p < 0.0001) and in OS (log-rank test, p = 0.002). In the adjusted analysis, we found that higher grade was generally associated with increased HRs, indicating worse outcomes. The c-index for DR, TR, and OS was 0.71, 0.69, and 0.67, respectively, suggesting moderate discriminative performance of the multivariable model, comparable to that of the Stratipath risk groups multivariable model.

## 4 Discussion

For our primary endpoint, DR, we found that the Stratipath high-risk group had a significantly higher HR of 2.01, indicating that the risk was doubled compared to the low-risk group within the first five years. For our secondary endpoint, TR, we observed similar results. These findings are consistent with the progression-free survival (PFS) reported by Sharma et al. [24], who observed a HR of 2.76 (95 % CI: 1.63–4.66). No significant HR differences were found among NHG2 patients for DR, TR, or OS when stratified into Stratipath high and low-risk categories. This lack of statistical significance may be due to a small effect size, insufficient sample size, or a combination. However, when using the more detailed Stratipath risk group variable (groups 1–5), we observed that higher risk groups were associated with increased HRs for DR and TR (p = 0.05), indicating a pattern of increasing relative event rates across the risk groups not captured by the binary high/low categorization.

In comparison with the previous results [24], we observe a more modest performance of the Stratipath high-vs. low-risk groups, with a HR for TR in all ER+/HER2-negative patients of 1.91 (95 % CI: 1.49–2.44, p = 0.0002) within the first five years. Possible explanations are the difference in endpoint definitions between PFS and TR, or that we used a competing risk analysis, which accounts for the fact that competing events can prevent the event of interest from occurring, providing a more conservative risk estimation. A key strength is the long follow-up, which is particularly important for postmenopausal patients with ER-positive/HER2-negative disease. This cohort is not directly comparable with the population included in previous validation studies [19,24], since our patients did not receive chemotherapy.

A limitation of this study is that the patients were diagnosed approximately 25 years ago, making direct comparisons to contemporary patients challenging due to differences in lifestyle, overall health status and clinical management. Nevertheless, these findings provide valuable insight into the natural history of breast cancer prognosis, offering important data that can inform future validation studies.

The current NHG system has notable limitations, primarily due to its inconsistency and high variability across laboratories, pathologists, and even within repeated assessments by the same pathologist [4,25]. This is suboptimal for patient care, as treatment decisions may be influenced by the laboratory or timing of evaluation. A comparison of the c-index between the Stratipath five-risk group models and the NHG model (Table 2) demonstrates similar discriminative ability for the endpoints DR, TR, and OS. However, the NHG model shows a slightly higher c-index for DR.

**Table 2:** Multivariable analysis for DR, TR and OS.

Variable	Year	DR		TR		OS	
		HR (95 % CI)	p-value	HR (95 % CI)	p-value	HR (95 % CI)	p-value
Stratipath risk category			<0.0001∗		0.0002∗		0.02∗
low		Reference		Reference		Reference	
high	0–5	2.01 (1.49–2.71)		1.91 (1.49–2.44)		1.35 (1.05–1.73)	
	5–10	1.27 (0.86–1.87)		1.20 (0.86–1.68)			
	5–21					1.11 (0.98–1.24)	
		c-index = 0.70		c-index = 0.69		c-index = 0.66	
Stratipath risk group (cat.)			<0.0001∗		<0.0001∗		<0.005∗
1		Reference		Reference		Reference	
2		1.91 (1.17–3.13)		1.84 (1.17–2.88)		1.10 (0.94–1.30)	
3		2.63 (1.63–4.24)		2.36 (1.52–3.67)		1.28 (1.08–1.50)	
4		3.18 (2.00–5.07)		2.78 (1.81–4.27)		1.32 (1.12–1.55)	
5		3.25 (2.00–5.28)		2.95 (1.89–4.61)		1.20 (1.00–1.44)	
		c-index = 0.71		c-index = 0.68		c-index = 0.66	
Nottingham Grade			<0.0001∗		<0.0001∗		<0.0001∗
1		Reference		Reference		Reference	
2	0–5	2.65 (1.62–4.33)		2.40 (1.53–3.76)		1.41 (1.02–1.97)	
	5–10	2.25 (1.62–4.33)		1.42 (0.91–2.20)			
	5–21					1.14 (1.00–1.31)	
3	0–5	6.65 (3.92–11.27)		5.59 (3.42–9.14)		2.73 (1.85–4.04)	
	5–10	1.69 (0.85–3.38)		1.50 (0.80–2.82)			
	5–21					1.20 (0.98–1.46)	
		c-index = 0.72		c-index = 0.69		c-index = 0.67	
Stratipath risk category (NHG2 patients subgroup analysis)			0.31		0.27		0.84
low		Reference		Reference		Reference	
high	0–5	1.33 (0.91–1.96)		1.34 (0.93–1.94)		1.35 (1.05–1.73)	
	5–10	1.12 (0.69–1.80)		1.11 (0.70–1.76)			
	5–21					1.11 (0.98–1.24)	
		c-index = 0.70		c-index = 0.68		c-index = 0.66	
Stratipath risk group (cat., NHG2 patients subgroup analysis)			0.05∗		0.05∗		0.31
1		Reference		Reference		Reference	
2		1.08 (0.59–1.97)		1.27 (0.71–2.28)		0.98 (0.79–1.23)	
3		1.85 (1.06–3.20)		1.97 (1.14–3.40)		1.15 (0.92–1.44)	
4		1.73 (0.99–3.00)		1.82 (1.05–3.15)		1.17 (0.94–1.45)	
5		1.73 (0.94–3.18)		2.00 (1.11–3.62)		1.04 (0.81–1.34)	
		c-index = 0.71		c-index = 0.69		c-index = 0.67	

Table 2. Multivariable analysis including only the main variables of interest. Baseline variables: age, lymph nodes, ER-status and tumorsize, were included in all models, but are not reported in this table. The variables Stratipath risk category, Stratipath risk group (categorical), Stratipath risk group (continuous), and NHG have been interchanged in different multivariable models for DR, TR, and OS, as described further in the methods section.

∗statistically significant.

Previous DL models for histopathology tasks have shown several issues with lack of diagnostic accuracy [26,27], or moderate to poor agreement between pathologist evaluation and DL-models [28], possibly due to lack of robustness, if developing DL-models using weakly supervised labels on WSI-levels as ground truth on too small dataset [10]. Although AI has shown strong diagnostic potential, thorough evaluation of its performance is warranted [26,29].

A study developing a deep learning model for malignancy grading found that incorporating non-tumorous elements, such as immune, stromal, and spatial features, improved the prediction of survival outcomes, exceeding the performance of pathologists using the NHG system [30]. Another study showed that stromal elements alone can identify invasive from benign breast cancer tissue in radiology images [31]. Incorporating some of these features into the Stratipath risk group grading model could provide a valuable perspective and potentially enhance its performance beyond that of pathologists using the NHG system.

In on our analysis of DR, risk groups 3–5 showed converging survival curves after ∼7.5 years, with groups 4 and 5 exhibiting similar adjusted HRs, suggesting a potential plateau effect among higher-risk patients. This pattern, consistent with the univariable results, suggest that while early HR differences, risk group 3 catches up over time. As the cohort included ER + HER2-negative patients not receiving chemotherapy, future studies should investigate whether risk group 3 might benefit from more aggressive treatment, under current standards of oncological treatment. In the discordant subgroups, notable patterns emerged with respect to intrinsic subtype distribution. Most patients classified as discordant low (NHG3/Stratipath group 1) were luminal A, whereas most patients classified as discordant high (NHG1/Stratipath group 5) were luminal B. Although these analyses are exploratory and based on small numbers, the findings suggest that the Stratipath Breast algorithm may identify morphological features that reflect underlying tumor biology in a manner similar to molecular assays such as PAM50 (Prosigna).

In this study, the prognostic performance of Stratipath Breast was independently validated in a separate cohort with long-term follow-up. The five-risk group AI model demonstrated prognostic performance comparable to the NHG system. Unlike the NHG system, which is influenced by inter-assessor variability, Stratipath Breast applied a standardized model-based approach that enables more granular risk stratification (risk groups 1–5). However, consistency across different pathology slides still warrants further evaluation. As AI-based decision support systems do not require complex laboratory setups, they offer the potential for more reproducible, scalable and biologically informative risk stratification. We observed that patients in discordant low- and high-risk groups tended to display intrinsic subtypes more consistent with the Stratipath 5-risk group classification than with Nottingham Grading. However, the number of cases and events was insufficient to determine whether these differences translated into survival outcomes. This clinical perspective merits further investigation in future studies.

## Sex and gender considerations

This study was conducted on data from female patients with breast cancer. As male breast cancer cases are rare and were not represented in the dataset, sex-based comparative analyses were not performed. While the model was trained and validated on female data, the underlying methodology may be applicable to male breast cancer with appropriate future validation. Sex was defined based on clinical records; gender identity was not assessed.

## CRediT authorship contribution statement

**Sandra Sinius Pouplier:** Writing – original draft, Project administration, Methodology, Investigation, Funding acquisition, Formal analysis, Data curation, Conceptualization. **Abhinav Sharma:** Writing – review & editing, Data curation. **Pekka Ruusuvuori:** Writing – review & editing, Supervision, Methodology, Conceptualization. **Johan Hartman:** Writing – review & editing, Methodology, Data curation, Conceptualization. **Maj-Britt Jensen:** Writing – review & editing, Supervision, Methodology, Formal analysis. **Bent Ejlertsen:** Writing – review & editing, Supervision, Methodology. **Mattias Rantalainen:** Writing – review & editing, Supervision, Software, Methodology, Conceptualization. **Anne-Vibeke Lænkholm:** Writing – review & editing, Supervision, Project administration, Methodology, Funding acquisition, Conceptualization.

## Ethics approval

This study was conducted in accordance with the 1964 Declaration of Helsinki and its later amendments. Ethical approval was obtained from the appropriate ethics committees, including the Regional Committee on Health Research Ethics for Region Zealand (Approval number: SJ-986).

## Declaration of generative AI in scientific writing

During the preparation of this work, the authors used CPT-4 in order to enhance the language and readability. After using this tool/service, the authors reviewed and edited the content as needed and take full responsibility for the content of the publication.

## Funding

This work was supported by the Innovation Fund Denmark, the Danish Cancer Research Fund, the Nordic Cancer Union, the Region Zealand Health Research Fund, and a 10.13039/501100009457Region Zealand PhD stipend. The funding sources had no role in the study design, data collection, analysis or interpretation, manuscript preparation, or the decision to submit the article for publication.

## Declaration of competing interest

JH reports speaker honoraria or advisory board remunerations from Sakura, Novartis, AstraZeneca, Pfizer, Eli Lilly, MSD, and Gilead, as well as institutional research support from Roche, MSD and Novartis. MR and JH are co-founders and shareholders of Stratipath AB. AS is employed by Stratipath AB and hold employee stock options. MJ reports serving on advisory board for Novartis. PR is co-founder & shareholder in Louhi Health Data company. AL reports receiving an institutional grant from AstraZeneca; serving on advisory boards for MSD and AstraZeneca; and receiving travel expenses from Daiichi Sankyo and AstraZeneca. BE reports outside the submitted Institutional grants from AstraZeneca, Daiichi Sankyo, Eli Lilly, Gilead, Novartis, Pfizer, and Seagen; Travel and Accommodation Expenses from: Daiichi Sankyo, MSD, and Pfizer. All other authors declare no conflicts of interest.
