# Supplementary material for: Validation of a deep learning-based AI model for breast cancer risk stratification in postmenopausal ER+/HER2-breast cancer patients
Source: Breast. 2025 Dec 4;85:104671. doi: 10.1016/j.breast.2025.104671 (PMC12756034; doi:10.1016/j.breast.2025.104671)
Supplement: Multimedia component 1 [file mmc1.docx]

**Supplementary results**:

**Table 1C. Distribution of NHG across Stratipath risk groups**

| **Variable** | **Nottingham Histological Grade, n (%)** | | | **Total** | **p-value** |
| --- | --- | --- | --- | --- | --- |
|  | 1 (n = 666) | 2 (n = 1493) | 3 (n = 307) | n = 2466 |  |
| Stratipath risk group |  |  |  |  | < 0.0001 |
| 1 | 265 (39.8) | 220 (14.7) | 8 (2.6) | 493 (20.0) |  |
| 2 | 179 (26.9) | 352 (23.6) | 19 (6.2) | 550 (22.3) |  |
| 3 | 124 (18.6) | 347 (23.2) | 36 (11.7) | 507 (20.6) |  |
| 4 | 78 (11.7) | 374 (25.1) | 99 (32.2) | 551 (22.3) |  |
| 5 | 20 (3.0) | 200 (13.4) | 145 (47.2) | 365 (14.8) |  |

Figure 3. CIF-plot for DR, ER groups

Figure 3: CIF plot for DR, ER groups 10-59, 60-89 and 90-100%. Grays test: p = 0.013
